# Spanish and Catalan Versions of the eHealth Literacy Questionnaire: Translation, Cross-Cultural Adaptation, and Validation Study

**DOI:** 10.2196/49227

**Published:** 2024-05-10

**Authors:** Eulàlia Hernández Encuentra, Noemí Robles, Ariadna Angulo-Brunet, David Cullen, Ignacio del Arco

**Affiliations:** 1 eHealth Center Universitat Oberta de Catalunya Barcelona Spain; 2 Faculty of Psychology and Education Universitat Oberta de Catalunya Barcelona Spain; 3 Language Service Universitat Oberta de Catalunya Barcelona Spain

**Keywords:** eHealth literacy, eHealth, digital health, health literacy, questionnaire, eHealth Literacy Questionnaire, eHLQ, validation

## Abstract

**Background:**

The rise of digital health services, especially following the outbreak of COVID-19, has led to a need for health literacy policies that respond to people’s needs. Spain is a country with a highly developed digital health infrastructure, but there are currently no tools available to measure digital health literacy fully. A well-thought-through questionnaire with strong psychometric properties such as the eHealth Literacy Questionnaire (eHLQ) is important to assess people’s eHealth literacy levels, especially in the context of a fast-growing field such as digital health.

**Objective:**

This study aims to adapt the eHLQ and gather evidence of its psychometric quality in 2 of Spain’s official languages: Spanish and Catalan.

**Methods:**

A systematic cultural adaptation process was followed. Data from Spanish-speaking (n=400) and Catalan-speaking (n=400) people were collected. Confirmatory factor analysis was used to confirm the previously established factor structure. For reliability, the Cronbach α and categorical ω were obtained for every subscale. Evidence of convergent and discriminant validity was provided through the correlation with the total score of the eHealth Literacy Scale. Evidence based on relations to other variables was evaluated by examining extreme values for educational level, socioeconomic level, and use of technology variables.

**Results:**

Regarding the confirmatory factor analysis, the 7-factor correlated model and the 7 one-factor models had adequate goodness-of-fit indexes for both Spanish and Catalan. Moreover, measurement invariance was established between the Spanish and Catalan versions. Reliability estimates were considered adequate as all the scales in both versions had values of >0.80. For convergent and discriminant validity evidence, the eHealth Literacy Scale showed moderate correlation with eHLQ scales in both versions (Spanish: range 0.57-0.76 and *P*<.001; Catalan: range 0.41-0.64 and *P*<.001). According to the relationship with external variables, all the eHLQ scales in both languages could discriminate between the maximum and minimum categories in level of education, socioeconomic level, and level of technology use.

**Conclusions:**

The Spanish and Catalan versions of the eHLQ appear to be psychometrically sound questionnaires for assessing digital health literacy. They could both be useful tools in Spain and Catalonia for researchers, policy makers, and health service managers to explore people’s needs, skills, and competencies and provide interesting insights into their interactions and engagement regarding their own experiences with digital health services, especially in the context of digital health growth in Spain.

## Introduction

### Background

The COVID-19 pandemic has accelerated the development of digital health infrastructures, devices, and services. Different digital health solutions were used in different settings worldwide with different approaches and purposes regarding, for example, the dissemination of public information for prevention, the understanding of the disease, the generation of diagnostic tools, or the sharing of real-time data for surveillance and control of the spread of the infection [1]. This scenario provided an opportunity to develop and test the capacity of digital technologies to increase the quality of health services, which required a similar improvement in citizens’ digital skills. This is reflected in the World Health Organization global strategy on digital health 2020-2025 [2], one of whose 4 strategic objectives is to advocate for people-centered digital health systems. To this end, it is necessary to promote adequate health literacy with regard to digital health services among users and health care professionals to encourage the adoption and active use of these services.

In line with the World Health Organization strategy, and as other countries such as Ireland, New Zealand, the United Kingdom, and the United States had previously done [3-6], the Spanish Ministry of Health established, as part of its Digital Strategy 2021-2026, that one of the strategic objectives would be the empowerment and involvement of people in their health care and disease control by facilitating the relationship with health services [7]. Within this framework, people’s trust in the health system is established as the key element for the implementation of a digitalized and efficient health system [8,9].

In general terms, data from recent reports and surveys show that Spain can be considered one of the leading countries in the use of digital technologies related to health. This is also the case in Catalonia, a region in northeastern Spain with almost 10 million Catalan speakers [10]. According to Eurostat [11], almost 7 out of 10 Spanish citizens used the internet to seek health information in 2021 (European Union average 56%), which is close to the leading countries in the area (Finland: 80%; the Netherlands: 77%; Norway: 77%; Denmark: 75%). In fact, digital clinical appointments were already available in 40% of Spain in 2019 (51.7% in Catalonia), in second position behind Finland (53%). In addition, in 2021, digital medical records and electronic prescriptions were widely implemented (with different levels of access and services depending on the region). A European survey on digital health across Europe [8] showed that Spain outperforms the average of the countries surveyed in indicators such as digital clinical consultations, the use of mobile health apps, access to electronic medical records, and remote supervision for monitoring or sharing symptoms with health professionals, with only 25% of Spanish people not using any digital technology to manage their health in the last year (a proportion that rises to 36% in the rest of the world) [9]. However, the use of wearables for health monitoring together with digital access for mental health in Spain show lower indicators than those of the rest of the countries studied. These promising data are complemented by the positive attitude of Spanish citizens toward the adoption of digital health even among those who have not yet used it [8].

The development of digital health services must be accompanied by digital health literacy, skills, confidence, and a positive attitude toward the relevance of these services [12-15] so that they can respond to people’s needs in an equitable and inclusive way. Understanding these needs has been difficult due to the lack of a rigorous theoretical framework for measuring digital health literacy [16]. In 2006, Norman and Skinner [12] pioneered the development of their eHealth Literacy Scale (eHEALS) to assess individuals’ self-perceived ability to seek, find, understand, and evaluate health information from electronic sources and apply the knowledge gained to address a problem. Currently, there are different instruments available to measure digital health literacy [17]. However, the only tools currently available in Spanish are the eHEALS questionnaire [12] and the European Health Literacy Survey Questionnaire (HLS-EU-Q) [18], an instrument developed by the European Health Literacy Consortium [19]. However, the HLS-EU-Q is designed primarily to assess health literacy and not digital health literacy. The short version of the HLS-EU-Q (16 items) includes 3 items that assess the ability to evaluate reliability, protect oneself, and understand the health information available in the media (including on the internet). However, this is clearly insufficient in a digital health context. Similarly, as with the 3 items in the short version of the HLS-EU-Q, the eHEALS questionnaire does not capture the breadth of services and aspects that digital health now covers [17]. Moreover, neither instrument is available in Catalan.

To address the shortcomings of the eHEALS in moving beyond Health 1.0, the Digital Health Literacy Instrument was developed, a performance-based tool aiming to include the interactivity of the web [20]. However, it did not comprehensively capture the interactions that digital health entails, including aspects linked to individuals (information and knowledge about one’s own health), the intersection between users and technologies (the feeling of being safe and in control and their motivation), and users’ experience of the systems (that they work and are accessible and that they respond to users’ needs). To cover all these aspects, the eHealth Literacy Questionnaire (eHLQ) was developed [21,22]. The eHLQ is an instrument with 35 items distributed in 7 scales that each assess a dimension of digital health literacy (Textbox 1).

**eHealth Literacy Questionnaire scales, description, and number of items.**

**Questionnaire overview**

- Scale 1 (using technology to process health information; 5 items): being able to use technologies to read, write, remember, appraise, and apply health information
- Scale 2 (understanding of health concepts and language; 5 items): being able to know and understand how the body functions, the current health status, and risks
- Scale 3 (ability to actively engage with digital services; 5 items): being comfortable using digital services for handling information
- Scale 4 (feel safe and in control; 5 items): feeling confident about safety, access, and storage of personal health data
- Scale 5 (motivated to engage with digital services; 5 items): feeling that engaging in the use of digital services will be useful for health management
- Scale 6 (access to digital services that work; 6 items): having access to digital services that work when the users need them and in the way they expect them to work
- Scale 7 (digital services that suit individual needs; 4 items): having access to responsive and adaptable services so that they suit the users’ needs

Each scale has between 4 and 6 items with 4 response options (*strongly disagree*, *disagree*, *agree*, and *strongly agree*) with an assigned value of 1 to 4. With the aim of describing the person’s relationship with digital health services in a broad way, the questionnaire does not have a total score. This is because health literacy is a relational concept [23], and it is firmly rooted in a specific context, so it depends on the characteristics of each health system. Furthermore, to make it user-friendly, the questionnaire is designed to allow for modular use (by using isolated scales) depending on the aspects that are of interest to analyze in each context (eg, people’s ability to use health services or the availability of services that respond to their needs). It is because of this descriptive intent that, rather than quantitative scores, the eHLQ analysis results in user profiles from which vignettes (scenarios) can be defined that exemplify people’s needs, skills, and competencies and provide insights into their interactions and engagement regarding their own experiences with digital health services. These can be used by policy makers and health service managers to design and offer improved services.

The eHLQ was constructed simultaneously in Danish and English to minimize cultural references [22]. It has shown good internal consistency reliability, and beyond these 2 original versions, as far as the authors of this study are aware, there are 3 more published versions available today that have shown positive evidence of psychometric quality ([24] in Mandarin [25], Swedish [26], and Dutch), although other adaptations are in progress across Europe. The eHLQ has been used to assess health literacy in different groups [27-29] both in Denmark and Australia [27,30], and further extensions of it have been developed for application in specific groups, for example, for health professionals [31] or older people, to assess their readiness to use digital health services [32]. Therefore, it is constantly evolving and a suitable tool to be used in a context of digital health growth such as that in Spain.

### Objectives

The main objective of this study was to undertake the translation and cultural adaptation of the eHLQ and gather evidence of psychometric quality of the eHLQ scores in a large sample of the general population in Spain, creating a simultaneous process for 2 of the official languages spoken in the country: Spanish and Catalan.

## Methods

### Study Design

**Figure 1:**
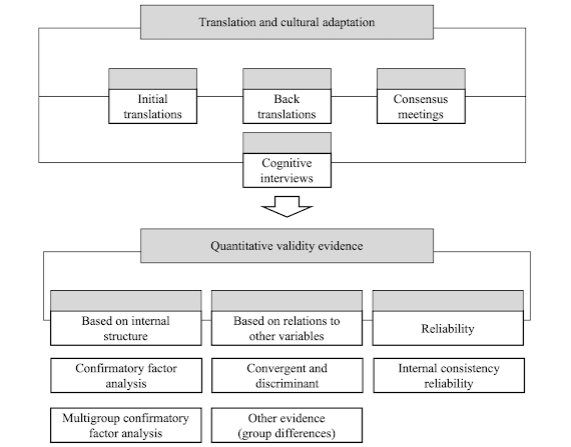
Schematic overview of the study design.

This study involved the translation and cross-cultural adaptation of the eHLQ into Spanish and Catalan. A cross-sectional design was used to gather evidence of the psychometric quality of the Spanish and Catalan versions of the eHLQ (Figure 1).

### Translation and Cultural Adaptation

The translation and cultural adaptation process followed version 5 of the Translation Integrity Procedure [33], which provides systematic translation documentation and helps adaptation teams maximize measurement equivalence [34]. The same process was used for both the translation into Spanish and into Catalan and consisted of three main steps:

1. A professional translator produced an initial translation, which was revised by a second professional translator. In each case, the translators were fluent in English and native speakers of Spanish and Catalan. The translators were provided with the eHLQ in English and detailed information on the intent of each of the items.
2. In total, 2 professional translators (fluent in Spanish and Catalan and native speakers of English) then produced blinded back translations.
3. A series of consensus meetings were then held to critically review the proposed translations. These meetings involved the forward translators, the authors of this paper, and the authors of the eHLQ. These meetings analyzed the translations (with the help of the back translations for those who were not fluent in Catalan or Spanish), the cultural adaptation, and the equivalence of items with the original English version. Each item was examined in turn to confirm that (1) the intent of the translation matched that of the original; (2) the translations were appropriate across gender and education categories, dialects, and levels of digital health literacy; and (3) people would find it equally easy to respond (*strongly disagree*, *disagree*, *agree*, and *strongly agree*) to the translations as they would to the questions in English.

Cognitive interviews were carried out with 8 Spanish speakers and 8 Catalan speakers (12/16, 75% female; aged 22 to 81 years; 3/16, 19% with a primary school education; 5/16, 31% with a high school education; 8/16, 50% with a university education; 3/16, 19% with a rare or occasional use of technology; 6/16, 38% with a medium use of technology; and 7/16, 44% with habitual or continuous use of technology) to field-test the intelligibility and equivalence of each translation. Following a thinking-aloud approach, respondents were asked to answer each item after silently reading it. Scripted probes were used to explore difficulties and items that needed rephrasing or further explanation. Spontaneous probes were also used, for example, when the respondent hesitated or took a long time to answer an item. Changes suggested were introduced and discussed during the final consensus meeting to obtain the definitive versions of the questionnaire.

### Quantitative Validity Evidence

#### Sample

All data were collected anonymously. The sample was stratified by geographical area, gender, and age of the Spanish and Catalan populations. Inclusion criteria were being aged ≥18 years, being a Spanish or Catalan speaker (depending on the version), and being able to read and understand a text in one of those languages. The final sample comprised 800 adult respondents for the Spanish and Catalan versions (n=400, 50% for each version). Demographic data included age, gender, educational level, socioeconomic status, frequency of technology use, and self-reported health status.

#### Recruitment and Data Collection

Between May 2021 and May 2022, participants were recruited through an independent partner company that specializes in data capture and processing, statistics, and market research. With >50 years of experience, the company [35] offers high-quality data collection and assessment services and has access to panels of respondents sourced through different methods to reach a wider variety of profiles (eg, loyalty programs in the travel, leisure, and retail sectors; banners, bloggers, influencers, and in-app advertising; and social networks and web-based communities) who had previously registered to participate as volunteers in different studies. The recruitment company targeted a potential 1,080,000 participants eligible for the Spanish sample and 140,000 participants eligible for the Catalan sample who were likely to qualify based on the demographic characteristics indicated in their user profiles. Gender, age group, and place of residence were used to ensure stratified samples mirroring the Spanish and Catalan populations. The company randomly contacted potential participants directly, with a detailed explanation of the project, the informed consent document, and an invitation to participate in a computer-aided web interview including a self-administered version of the eHLQ. Responses were accepted until balanced quotas were reached. To reach 300 responses for each language, the company contacted 350 potential participants for the Spanish sample and 352 for the Catalan sample. High response rates were achieved in both samples (300/350, 85.7% and 300/352, 85.2% for the Spanish and Catalan samples, respectively); thus, no reminders were sent, and no further potential participants were contacted.

As the digital panel was not able to ensure representativity of older people or people with low educational levels or digital skills, a further 200 responses (n=100, 50% for each language) were added to the samples. These respondents were recruited through direct approach in a wide range of settings in the community (health and social services and parks). For this subset of the sample, trained recruiters explained the objectives of the research, and after checking the basic requirements of gender and age group, they provided support to the participants to fill out a version of the same questionnaires on a tablet.

All the instruments were digitalized using COMPLET (ODEC), which is proprietary software [35]. It is used for in-person and digital interviews and surveys, and saves the data on their own servers.

Filling out the questionnaires took approximately 15 minutes. The questionnaires were self-administered or, in the subset of the sample recruited in community settings and where necessary, trained recruiters provided support.

The authors monitored both the recruitment and data gathering processes carried out by the partner company by providing the definition of the pool and sampling criteria, reviewing the digitalization process, and giving specific training instructions to recruit and survey the subset sample reached in community settings. Finally, an initial attention check (ie, an initial survey item that required respondents to provide a specific response) and a speeding check (ie, respondents whose survey duration was less than one-third of the mean survey duration) were asked to be included to guarantee data quality. Those who did not pass either quality control round were excluded from the final samples.

#### Instruments

The Spanish and Catalan versions were created from the eHLQ questionnaire [22] (refer to the Translation and Cultural Adaptation section). For convergent validity evidence, as with previous studies [36], a positive correlation was assumed between the first scale of the eHLQ and the eHEALS. Evidence of discriminant validity with the rest of the eHLQ subscales was also provided. As no other instrument was available in Spanish or Catalan for digital health literacy, the Spanish version [37] of the eHEALS questionnaire [12] was used as it has shown positive evidence of psychometric quality.

Self-categorization was also used, with a single self-reported question for technology use [38] and a single self-reported question about health status.

Educational level was collected in one self-reported question, and socioeconomic status was calculated according to a reviewed index based on a set of questions that take into account not only income but also education; job; housing; and financial aid for the family unit, not only for the respondent [39].

#### Statistical Analysis

Stata (version 17.0; StataCorp) and the R software (R Foundation for Statistical Computing) were used for the statistical analysis. Regarding the descriptive analysis, means and SDs were calculated for quantitative variables, and frequencies were calculated for categorical variables. To test differences for the Spanish and Catalan samples, we used 2-tailed *t* tests for quantitative variables and the chi-square test for categorical variables.

For the quantitative evidence, we partially followed the methodology used by previous researchers [15]. Following the recommendations of Doval et al [40], and considering that a 4-point Likert scale was used, the weighted least squares mean and variance corrected estimator was used for the confirmatory factor analysis (CFA). To assess goodness-of-fit indexes (GOFIs), the criteria by Hu and Bentler [41] were followed, which consider adequate a comparative fit model (comparative fit index [CFI]), a Tucker-Lewis index (TLI) of >0.90, and a root mean square error of approximation (RMSEA) and standardized root mean square residual of <0.05. CFA was used to confirm the established factor structure. Seven 1-factor models and a 7-factor model were fitted. Metric invariance (same factor loadings) was assessed, and thresholds were also set to be equal between both groups. To do so, a change of <0.01 in CFI and RMSEA was also considered [42].

As for internal consistency reliability, the Cronbach α and categorical ω were provided for every subscale [40]. The categorical ω coefficient was reported from the 1-factor models. To assess reliability, a value of >0.80 was considered adequate for research purposes, and a value of 0.90 was considered adequate for individual decisions [43].

Given the approximately normal distribution of test scores (skew and kurtosis of <1 point in the Spanish and Catalan samples), the correlation among the 7 scales of the eHLQ was assessed through a Pearson correlation matrix. Convergent and discriminant validity evidence was assessed using the eHEALS and was evaluated by correlating the total score of the eHEALS with the score of each eHLQ scale.

Finally, the relationship with external variables was evaluated through the association between the eHLQ scores in each scale with the educational and socioeconomic variables and the use of digital technology variables. However, only the minimum and maximum categories were considered: primary school vs university for educational level, a high level corresponding to A1 or A2 vs a low level corresponding to E1 or E2 for socioeconomic level, and never to occasionally vs continuous use for the use of digital technology). The *t* test or a nonparametric test was used for the comparison according to the normal distribution of each eHLQ domain. Measurement invariance across groups could not be assessed due to sample size.

For the calculation of the initial sample, it was deemed necessary to have a minimum of 10 participants per item to conduct the factorial analyses (35 × 10 = 350). It was decided to slightly surpass that number and reach 400 participants. Finally, a post hoc analysis was conducted using *semPower* [44]. For a sample size of 400, an α error of .05, and expecting an RMSEA of 0.05, there was a power of >0.999.

### Ethical Considerations

Ethics approval was obtained from the Ethics Committee of the Universitat Oberta de Catalunya (20201013_ ehernandez_Traduccio), and the license for translating the eHLQ was obtained from Swinburne University of Technology, Australia (November 11, 2020).

All participants in the validation process gave informed consent before data collection and received a gift card for digital marketplaces as compensation. Data were gathered anonymously to ensure participants’ confidentiality.

## Results

### Translation and Cultural Adaptation

During the series of consensus meetings, which involved the forward translators, the authors of this paper, and 2 of the authors of the original versions of the eHLQ, all the items for both the Spanish and Catalan translations were examined one by one. The first meeting served to highlight the most relevant points and pressing issues, which included the following agreements, all of which were applicable to both versions: (1) “health technology systems” and “eHealth systems” could be translated together as “digital health services”; (2) the “I find that...” expression at the beginning of different items was equivalent to “I think that...” but could not be removed and substituted by a more factual and objective form as it was essential to keep the point of view of the respondent; (3) “measurements” had a broader meaning than results from medical tests as it included other data sources, such as fitness-tracking devices; (4) the “works together” expression implied really interoperable systems, not just connected systems; (5) “find” could be translated as “search” (in relation to health information searching); and (6) it was not possible to combine ≥2 words together (eg, “data/measurements”) as it was key to the questionnaire that there be no ambiguity in terms of the intent of each item.

Throughout the systematic process of translation and cultural adaptation, the issues discussed were generally the same for both the Spanish and Catalan versions. In addition to the aforementioned agreements, a number of key wording points were addressed and revised, for example, “perfectly” versus “very well,” “being certain” versus “being sure,” “available” versus “at the disposal of,” “skills” versus “capabilities,” or simply word order according to linguistic and cultural uses. A few specific issues also arose from cognitive interviews that required rephrasing both in the Spanish version (Table 1) and the Catalan version (Table 2). In almost all cases, the issues were the same. Scale 4 (*Feel safe and in control*) did not need any changes after the cognitive interviews in either the Spanish or Catalan version.

Cognitive interviews raised the need to reinforce the information initially included in the original English version used. As well as adding a few more examples to the existing ones for health professionals, health technology, and digital health services, further information was included after issues were raised regarding items eHLQ26, eHLQ5, and eHLQ12.

Item eHLQ26 was particularly problematic as it was hard to express everything encapsulated in the word “measurements” with just one word in Spanish or Catalan. The possibility of using more than one word was ruled out as it is key to the questionnaire that there be no ambiguity in terms of the intent of each item. Adding “data” or “results” alongside “measurements” would open the door to respondents stating that they use one but not the other. In the end, the decision was made to translate “measurements about my body” as “data on how my body works.” In the instruction page, a new specific section was included to explain what “data on how my body works” meant by providing a few examples.

Similarly, items eHLQ5 and eHLQ12 raised the need to include a new communication and sharing information section on the instructions page, reinforcing the idea that having conversations or sharing information could be both informal and with professionals.

**Table 1:** Spanish version of the eHealth Literacy Questionnaire (eHLQ) and single-item changes after cognitive interviews.

Scale and item		First translation and first back translation	Final translation and back translation	Comments to changes
**Scale 1**				
	eHLQ11	Suelo usar la tecnología para entender problemas de salud. I’m used to using...	A menudo uso la tecnología para entender problemas de salud. I usually use...	Emphasizing frequency and avoiding any possible misunderstanding.
**Scale 2**				
	eHLQ5	Los conocimientos que tengo me permiten participar en conversaciones sobre temas de salud. ...conversations about health issues.	Mis conocimientos me ayudan a participar en conversaciones sobre temas de salud con otras personas. ...conversations about health issues with other people.	Including whom the conversations are with. Emphasizing that conversations are not only with health professionals but also with other people.
	eHLQ12	Tengo la información que necesito para hablar sobre mi salud. I have the information I need...	Dispongo de suficiente información para hablar sobre mi salud con otras personas. I have enough information...	Emphasizing whom the conversations are with.
	eHLQ26	Uso mis resultados sanitarios y otras mediciones sobre mi cuerpo para entender mi salud. I use my health results and other measurements...	Uso los datos sobre el funcionamiento de mi cuerpo para entender mi salud. I use data about how my body works...	Avoiding lists of items (results and measurements). Clarifying that health measurements (original) include professional and informal measurements. “Data” was the final option but specifying which data the item is referring to.
**Scale 3**				
	eHLQ32	Aprendo rápido a usar las novedades en tecnología para la salud. I learn quickly...	Aprendo fácilmente a usar las novedades en tecnología para la salud. I learn easily...	Sounding more natural (adverb change from “quickly” to “easily”). Promoting the idea of an ability as a continuous behavior.
**Scale 5**				
	eHLQ2	La tecnología me hace sentir que participo activamente en mi salud. Technology makes me feel that I am participating...	La tecnología me hace sentir que me implico en el cuidado de mi salud. Technology makes me feel involved...	Culturally sounding more natural (from actively participating to involvement).
**Scale 6**				
	eHLQ9	Los profesionales de la salud me ofrecen servicios a los que puedo acceder mediante la tecnología. Health professionals offer me...	Los profesionales de la salud que me atienden me ofrecen servicios a los que puedo atender mediante la tecnología. Health professionals who look after me offer me...	Emphasizing who the health professionals are.
	eHLQ16	Mis datos de salud están a mi disposición desde cualquier lugar. ...available to me from anywhere.	Mis datos de salud están a mi disposición dondequiera que yo esté. ...available to me from wherever I am.	Emphasizing the point of view of the respondent instead of data location.
	eHLQ34	Tengo a mi disposición tecnología para la salud que me funciona bien. I have access to health technology...	Tengo a mi disposicion servicios de salud digital que siempre funcionan. I have access to digital health services...	Clarifying what health technology really means in a broader sense (“digital health services”). Emphasizing that it always works as intended.
**Scale 7**				
	eHLQ28	Los servicios de salud digital parece que se adaptan a mis necesidades particulares. I think eHealth services seem to adapt to my particular...	Creo que los servicios de salud digital se adaptan a mis necesidades. I think that digital health services adapt to my...	Keeping the point of view of the respondent. Simplifying. No need to add the adjective “particular” to the needs. Unifying the expression “digital health services” used throughout the questionnaire.
	eHLQ31	Creo que me ofrecen servicios de salud digital que me resultan útiles. ...services that I find useful.	Creo que la forma en la que se me ofrecen los servicios de salud se adapta a mi. ...services are offered to me in a way that suits me.	Beyond usefulness and personalization, focusing not only on the health needs but also on the capabilities. Unifying the expression “digital health services” used throughout the questionnaire.

**Table 2:** Catalan version of eHealth Literacy Questionnaire (eHLQ) and single-item changes after cognitive interviews.

Scale and item			First translation and first back translation		Final translation and back translation		Comments to changes
**Scale 1**							
	eHLQ13	La tecnologia m’ajuda a decidir quina atenció mèdica em convé més. ...medical care is best for me.		La tecnologia m’ajuda a decidir quina atenció sanitaria em convé més. ...health care is best for me.		Clarifying the meaning as “health care” includes care from any health professional (not only medical doctors).	
**Scale 2**							
	eHLQ5	Els coneixements que tinc em permeten participar en converses sobre temes de salut. ...conversations about health matters.		Els meus coneixements m’ajuden a participar en converses sobre temes de salut amb altres persones. ...conversations about health issues with other people.		Including whom the conversations are with. Emphasizing that conversations are not only with health professionals but also with other people.	
	eHLQ26	Faig servir els meus resultats sanitaris I altres mesures sobre el meu cos per entendre la meva salut. I use my health results and other measurements...		Faig servir les dades sobre com funciona el meu cos per entendre la meva salut. I use data about how my body works...		Avoiding lists of items (results and measurements). Clarifying that health measurements (original) include professional and informal measurements. “Data” was the final option but specifying which data the item is referring to.	
**Scale 3**							
	eHLQ32	Aprenc ràpidament a fer server les novetats en tecnologia per a la salut. I learn quickly...		Aprenc fàcilment a fer server les technologies per a la salut. I learn easily...		Sounding more natural (adverb change from “quickly” to “easily”). Promoting the idea of an ability as a continuous behavior.	
**Scale 5**							
	eHLQ2	La tecnologia em fa sentir que participo activament en la meva salut. ...feel that I am participating actively...		La tecnologia em fa sentir que m’implico en la cura de la meva salut. ...feel involved...		Culturally sounding more natural (from actively participating to involvement).	
**Scale 6**							
	eHLQ9	Els professionals de la salut m’ofereixen serveis als quals puc accedir per mitjà de la tecnologia. Health professionals offer me...		Els professionals de la salut que m’atenen m’ofereixen servis de salut digital. Health professionals who look after me offer me...		Emphasizing who the health professionals are.	
	eHLQ16	Les meves dades de salut estan a la meva disposició des de qualsevol lloc. ...available to me from anywhere.		Les meves dades de salut estan a la meva disposició allà on jo sigui. ...available to me from wherever I am.		Emphasizing the point of view of the respondent instead of data location.	
	eHLQ34	Tinc a la meva disposició tecnologia per a la salut que funciona. ...health technology that works.		Tinc al meu abast serveis de salut digital que sempre funcionen. ...digital health services that always work.		Clarifying what health technology really means in a broader sense (“digital health services”). Emphasizing that it always works as intended.	
**Scale 7**							
	eHLQ28	Trobo que els serveis de salut digital s’adapten a les meves necessitats particulars. I think eHealth services seem to adapt to my particular...		Crec que els serveis de salut digital s’adapten a les meves necessitats. I think that digital health services adapt to my...		Simplifying. No need to add the adjective “particular” to the needs. Unifying the expression “digital health services” used throughout the questionnaire.	

### Quantitative Validity Evidence

#### Sample Description

Both samples had similar patterns of distribution (Table 3). Briefly, 51% (408/800) of the sample were women, and most of the participants were aged between 50 and 64 years (285/800, 35.6%), had a high school educational level (322/800, 40.3%) and a high-medium socioeconomic level (617/800, 77.1%), and lived in municipalities of >200,000 inhabitants (428/800, 53.5%). Regarding their health, they reported good health status, and they considered themselves habitual technology users.

**Table 3:** Demographic characteristics of the Spanish and Catalan samples (N=800).

			Spanish version (n=400)		Catalan version (n=400)		*P* value
**Gender, n (%)^a^**							>.99
	Woman	204 (51)		204 (51)			
	Man	196 (49)		196 (49)			
	I prefer not to answer	0 (0)		0 (0)			
**Age (y), n (%)^a^**							>.99
	18-34	81 (20.3)		81 (20.3)			
	35-49	103 (25.8)		103 (25.8)			
	50-64	142 (35.5)		143 (35.8)			
	≥65	74 (18.5)		73 (18.3)			
Age (y), mean (SD; range)^b^			49.22 (15.48; 18-79)		49.05 (15.80; 18-80)		>.99
**Educational level, n (%)^a^**							.43
	Primary school	97 (24.3)		90 (25.5)			
	High school	152 (38)		170 (42.5)			
	University	151 (37.8)		140 (35)			
**Socioeconomic index, n (%)^a^**							.83
	High (A1, A2, or B)	161 (40.3)		154 (38.5)			
	Medium (C or D)	147 (36.8)		155 (38.8)			
	Low (E1 or E2)	92 (23)		91 (22.8)			
**Inhabitants per municipality, n (%)^a^**							.96
	Up to 5000	26 (6.5)		24 (6)			
	5001-200,000	161 (40.3)		161 (40.3)			
	>200,000	213 (53.3)		215 (53.8)			
**Technology use, n (%)^a^**							.10
	Never or rarely	23 (5.8)		14 (3.5)			
	Occasionally	63 (15.8)		43 (10.8)			
	Medium	93 (23.3)		95 (23.8)			
	Habitual	131 (32.8)		153 (38.3)			
	Continuous	90 (22.5)		95 (23.8)			
**Health status, n (%)^a^**							.30
	Very bad	2 (0.5)		7 (1.8)			
	Bad	18 (4.5)		26 (6.5)			
	Regular	102 (25.5)		100 (25)			
	Good	229 (57.3)		214 (53.5)			
	Very good	49 (12.3)		53 (13.3)			
eHealth literacy (eHEALS^c^), mean (SD; range)^a^			27.35 (8.40; 8-40)		27.80 (7.08; 8-40)		.41

^a^Quantitative variable.

^b^Categorical variable.

^c^eHEALS: eHealth Literacy Scale.

#### CFA and Measurement Invariance

Table 4 shows the GOFIs for the proposed models. Table 5 shows standardized factor loadings for the proposed models.

The 7-factor correlated model showed adequate GOFIs (Table 4) for the Spanish and Catalan versions. Thus, in both cases, the fitting values were considered adequate. As can be seen, factor loadings were high and homogeneous (>0.60).

In turn, the seven 1-factor models had adequate GOFIs according to the CFI and TLI values in both versions of the questionnaire. RMSEA values were >0.08 for factor 5 in the Catalan version and for factors 1, 3, 5, and 6 in the Spanish version. This could be explained by the fact that factor loadings are high in those factors.

The results of multigroup CFA showed that there was configural invariance between both languages (χ^2^_1078_=2707.1; CFI=0.98; TLI=0.98; RMSEA=0.07), equal factor loadings (ΔCFI=0.001; ΔRMSEA=0.004), and equal factor loadings and thresholds (ΔCFI=0.007; ΔRMSEA=0.01). Thus, measurement invariance can be assumed for factor loadings but not for thresholds.

**Table 4:** Goodness-of-fit indexes for the proposed models.

Model		Chi-square (*df*)	*P* value	CFI^a^	TLI^b^	RMSEA^c^ (90% CI)	SRMR^d^
**Spanish**							
	7-factor model	1631.2 (539)	<.001	0.98	0.98	0.07 (0.07-0.08)	0.04
	Scale 1	103.9 (5)	<.001	0.99	0.98	0.23 (0.19-0.27)	0.02
	Scale 2	18.8 (5)	<.001	1.00	0.99	0.08 (0.05-0.13)	0.01
	Scale 3	23.7 (5)	<.001	1.00	1.00	0.10 (0.06-0.14)	0.01
	Scale 4	29.6 (5)	<.001	1.00	0.99	0.11 (0.08-0.15)	0.01
	Scale 5	40.3 (5)	<.001	1.00	0.99	0.14 (0.10-0.18)	0.01
	Scale 6	54.8 (9)	<.001	0.99	0.99	0.12 (0.09-0.15)	0.02
	Scale 7	3.9 (2)	.14	1.00	1.00	0.05 (0.00-0.12)	0.01
**Catalan**							
	7-factor model	1380.1 (539)	<.001	0.98	0.98	0.07 (0.06-0.07)	0.04
	Scale 1	32.9 (5)	<.001	0.99	0.99	0.12 (0.08-0.16)	0.02
	Scale 2	12.7 (5)	.03	1.00	0.99	0.06 (0.02-0.11)	0.02
	Scale 3	17.2 (5)	<.001	1.00	1.00	0.08 (0.04-0.12)	0.01
	Scale 4	17.3 (5)	<.001	1.00	1.00	0.08 (0.04-0.12)	0.01
	Scale 5	27.9 (5)	<.001	1.00	0.99	0.11 (0.07-0.15)	0.02
	Scale 6	19.9 (9)	<.001	1.00	1.00	0.06 (0.02-0.09)	0.01
	Scale 7	6.5 (2)	.04	1.00	1.00	0.08 (0.01-0.15)	0.01

^a^CFI: comparative fit index.

^b^TLI: Tucker-Lewis index.

^c^RMSEA: root mean square error of approximation.

^d^SRMR: standardized root mean square residual.

**Table 5:** Standardized factor loadings for the models of the Spanish and Catalan versions of the eHealth Literacy Questionnaire (eHLQ).

		7 factors	7 factors		1 factor	1 factor
		Spanish	Catalan	Spanish		Catalan
		Factor loading (95% CI)	Factor loading (95% CI)	Factor loading (95% CI)		Factor loading (95% CI)
**Scale 1: Using technology to process health information**						
	eHLQ7	0.88 (0.85-0.91)	0.89 (0.86-0.91)	0.90 (0.87-0.93)		0.86 (0.83-0.90)
	eHLQ11	0.82 (0.78-0.85)	0.89 (0.86-0.91)	0.92 (0.90-0.94)		0.86 (0.83-0.89)
	eHLQ13	0.90 (0.87-0.92)	0.92 (0.90-0.94)	0.87 (0.85-0.90)		0.89 (0.86-0.92)
	eHLQ20	0.85 (0.82-0.88)	0.87 (0.84-0.89)	0.88 (0.85-0.90)		0.85 (0.82-0.88)
	eHLQ25	0.91 (0.89-0.93)	0.91 (0.89-0.93)	0.88 (0.86-0.91)		0.86 (0.83-0.90)
**Scale 2: Understanding of health concepts and language**						
	eHLQ5	0.78 (0.74-0.83)	0.82 (0.78-0.86)	0.83 (0.79-0.87)		0.81 (0.76-0.87)
	eHLQ12	0.75 (0.70-0.80)	0.86 (0.83-0.88)	0.90 (0.87-0.92)		0.79 (0.74-0.84)
	eHLQ15	0.75 (0.70-0.80)	0.82 (0.78-0.86)	0.82 (0.78-0.85)		0.77 (0.71-0.82)
	eHLQ21	0.71 (0.65-0.76)	0.78 (0.73-0.82)	0.79 (0.75-0.83)		0.72 (0.67-0.78)
	eHLQ26	0.92 (0.89-0.95)	0.93 (0.91-0.96)	0.83 (0.80-0.87)		0.80 (0.76-0.85)
**Scale 3: Ability to actively engage with digital services**						
	eHLQ4	0.88 (0.85-0.91)	0.9 (0.88-0.93)	0.83 (0.79-0.87)		0.84 (0.81-0.88)
	eHLQ6	0.92 (0.89-0.94)	0.92 (0.90-0.94)	0.90 (0.87-0.92)		0.93 (0.90-0.95)
	eHLQ8	0.85 (0.81-0.88)	0.86 (0.84-0.89)	0.82 (0.78-0.85)		0.86 (0.83-0.90)
	eHLQ17	0.90 (0.87-0.92)	0.92 (0.89-0.94)	0.79 (0.75-0.83)		0.91 (0.87-0.94)
	eHLQ32	0.92 (0.89-0.94)	0.93 (0.91-0.95)	0.83 (0.80-0.87)		0.90 (0.90-0.92)
**Scale 4: Feel safe and in control**						
	eHLQ1	0.83 (0.78-0.87)	0.82 (0.78-0.86)	0.87 (0.84-0.90)		0.87 (0.84-0.90)
	eHLQ10	0.89 (0.86-0.93)	0.92 (0.89-0.94)	0.91 (0.89-0.93)		0.90 (0.88-0.93)
	eHLQ14	0.93 (0.89-0.97)	0.91 (0.87-0.94)	0.78 (0.74-0.82)		0.75 (0.70-0.80)
	eHLQ22	0.85 (0.81-0.89)	0.87 (0.84-0.90)	0.88 (0.85-0.91)		0.90 (0.87-0.92)
	eHLQ30	0.88 (0.84-0.91)	0.88 (0.85-0.92)	0.88 (0.85-0.91)		0.85 (0.81-0.88)
**Scale 5: Motivated to engage with digital services**						
	eHLQ2	0.85 (0.81-0.88)	0.88 (0.86-0.91)	0.87 (0.84-0.90)		0.85 (0.81-0.88)
	eHLQ19	0.90 (0.87-0.92)	0.92 (0.91-0.94)	0.92 (0.90-0.94)		0.90 (0.87-0.92)
	eHLQ24	0.82 (0.78-0.85)	0.88 (0.85-0.90)	0.88 (0.85-0.90)		0.82 (0.78-0.85)
	eHLQ27	0.85 (0.82-0.89)	0.90 (0.88-0.92)	0.90 (0.88-0.93)		0.84 (0.81-0.88)
	eHLQ35	0.87 (0.83-0.90)	0.91 (0.88-0.93)	0.89 (0.87-0.92)		0.86 (0.82-0.89)
**Scale 6: Access to digital services that work**						
	eHLQ3	0.78 (0.74-0.83)	0.81 (0.77-0.85)	0.76 (0.72-0.81)		0.78 (0.73-0.83)
	eHLQ9	0.72 (0.67-0.78)	0.77 (0.73-0.81)	0.78 (0.73-0.82)		0.72 (0.66-0.77)
	eHLQ16	0.83 (0.80-0.87)	0.78 (0.74-0.81)	0.78 (0.74-0.82)		0.80 (0.76-0.84)
	eHLQ23	0.79 (0.75-0.83)	0.88 (0.85-0.91)	0.88 (0.85-0.92)		0.80 (0.76-0.84)
	eHLQ29	0.84 (0.81-0.88)	0.85 (0.82-0.88)	0.84 (0.80-0.87)		0.84 (0.80-0.88)
	eHLQ34	0.87 (0.84-0.90)	0.90 (0.88-0.92)	0.91 (0.89-0.94)		0.86 (0.83-0.90)
**Scale 7: Digital services that suit individual needs**						
	eHLQ18	0.94 (0.92-0.96)	0.91 (0.89-0.93)	0.87 (0.84-0.89)		0.85 (0.81-0.88)
	eHLQ28	0.89 (0.87-0.92)	0.93 (0.91-0.95)	0.92 (0.90-0.94)		0.90 (0.88-0.92)
	eHLQ31	0.94 (0.92-0.95)	0.90 (0.88-0.92)	0.92 (0.90-0.94)		0.94 (0.93-0.96)
	eHLQ33	0.91 (0.89-0.93)	0.90 (0.88-0.92)	0.90 (0.88-0.92)		0.91 (0.89-0.93)

#### Mean Scores and Reliability

As can be seen in Table 6, all scales had a mean around 2.5 points, indicating that, on average, there was a good digital health literacy. All subscales had estimates of >0.80, which can be considered adequate for research purposes (Table 7). Moreover, except for scales 2 and 6, all scales had ω coefficients of >0.90, which is the minimum desirable threshold for making individual decisions. The values in the Spanish version, in general, were slightly higher than those in the Catalan version. As expected, considering that 1D factors were being assessed with high factor loadings, α and ω values were similar. Thus, all the internal consistency values were considered acceptable.

**Table 6:** Mean scores and internal consistency reliability for the Spanish and Catalan versions of the eHealth Literacy Questionnaire.

Scale	Spanish version			Catalan version		
	Values, mean (SD)	Cronbach α (95% CI)	ω (95% CI)	Values, mean (SD)	Cronbach α (95% CI)	ω (95% CI)
Using technology to process health information	2.41 (0.99)	0.90 (0.90-0.91)	0.92 (0.90-0.94)	2.55 (0.81)	0.91 (0.90-0.92)	0.91 (0.89-0.92)
Understanding of health concepts and language	2.60 (0.76)	0.85 (0.82-0.87)	0.87 (0.85-0.89)	2.65 (0.66)	0.85 (0.82-0.87)	0.85 (0.82-0.87)
Ability to actively engage with digital services	2.61 (0.97)	0.92 (0.91-0.93)	0.92 (0.90-0.93)	2.78 (0.79)	0.92 (0.91-0.93)	0.92 (0.90-0.9)
Feel safe and in control	2.65 (0.76)	0.90 (0.88-0.91)	0.90 (0.88-0.92)	2.72 (0.71)	0.90 (0.88-0.91)	0.90 (0.88-0.92)
Motivated to engage with digital services	2.46 (0.96)	0.90 (0.89-0.92)	0.92 (0.90-0.93)	2.54 (0.8)	0.90 (0.89-0.92)	0.91 (0.88-0.92)
Access to digital services that work	2.41 (0.82)	0.88 (0.87-0.89)	0.89 (0.87-0.91)	2.57 (0.71)	0.88 (0.87-0.90)	0.89 (0.86-0.90)
Digital services that suit individual needs	2.46 (0.97)	0.92 (0.91-0.93)	0.90 (0.88-0.92)	2.56 (0.83)	0.92 (0.91-0.93)	0.91 (0.88-0.93)

**Table 7:** Pearson correlation matrix between the Spanish (below the diagonal) and Catalan (above the diagonal) eHealth Literacy Questionnaire (eHLQ) scales and between the eHLQ scales and the eHealth Literacy Scale (eHEALS) score.

	eHEALS	Scale 1	Scale 2	Scale 3	Scale 4	Scale 5	Scale 6	Scale 7
eHEALS	1.00	0.63	0.64	0.60	0.41	0.60	0.59	0.61
Scale 1	0.76	1.00	0.86	0.84	0.66	0.89	0.83	0.83
Scale 2	0.71	0.83	1.00	0.83	0.65	0.82	0.79	0.79
Scale 3	0.76	0.89	0.82	1.00	0.67	0.82	0.80	0.81
Scale 4	0.57	0.68	0.74	0.69	1.00	0.71	0.78	0.71
Scale 5	0.74	0.91	0.82	0.87	0.73	1.00	0.85	0.89
Scale 6	0.70	0.86	0.80	0.84	0.76	0.88	1.00	0.88
Scale 7	0.76	0.90	0.82	0.89	0.73	0.93	0.89	1.00

#### Correlation Matrix

Concerning the correlations among the eHLQ scales, they ranged from 0.69 to 0.93 in the Spanish version and from 0.67 to 0.90 in the Catalan version (Table 7). In both versions, the scale with the lowest correlations with the other dimensions was scale 4 (*Feel safe and in control*). In the Spanish version, the highest correlation was between scales 5 and 7 (*Motivated to engage with digital services* and *Digital services that suit individual needs*; 0.93), and in the Catalan version, it was between scales 1 and 5 (*Using technology to process health information* and *Motivated to engage with digital services*; 0.90).

#### Convergent and Discriminant Validity Evidence

For gathering further evidence of discriminant and convergent validity, the eHEALS was administered. A high correlation was expected with the scale “Using technology to process health information,” and lower correlations were expected with the rest (discriminant validity evidence). Once analyzed, for the eHEALS, the mean value for the Spanish version was 27.35 (SD 8.40), and for the Catalan version, the mean was 27.80 (SD 7.08); in both versions, the range was from 8 to 40. The analysis of the eHEALS and the different eHLQ scales (Table 7) showed a moderate correlation of the global eHEALS score with the eHLQ scales of the Spanish version, with the correlations with scale 1 (*Using technology to process health information*; *r*=0.76), scale 3 (*Ability to actively engage with digital services*; *r*=0.76), and scale 7 (*Digital services that suit individual needs*; *r*=0.76) being the highest and the correlation with scale 4 (*Feel safe and in control*; *r*=0.57) being the lowest. Regarding the Catalan version, the values were slightly lower than those for the Spanish version. Again, scale 4 had the lowest correlation with the eHEALS (*Feel safe and in control*; *r*=0.41), whereas scale 2 had the highest correlation (*Understanding of health concepts and language*; *r*=0.64). Thus, while positive convergent evidence was achieved with scale 1, no positive discriminant evidence was found in the rest of the scales. This could be explained by the high correlation that all the scales of the eHEALS have.

#### Relationship With External Variables

The scores for all the eHLQ scales were lower in the group with a primary school education in comparison to the group with a university education both in the Spanish and Catalan versions. As measurement invariance could not be assessed due to sample size limitations, it is not possible to assess whether these differences are due to measurement invariance or a real impact. These differences were statistically significant in all the scales (Table 8); thus, both versions discriminated between these groups in all the scales.

The scores for all the eHLQ scales were lower in the group with a low socioeconomic level in comparison to the group with a high socioeconomic level both in the Spanish and Catalan versions. These differences were significant in all the scales (Table 9); thus, both versions discriminated between these groups in all the scales.

The scores for all the eHLQ scales were lower in those who never used technology or used it occasionally in comparison to those who reported a continuous use both in the Spanish and Catalan versions. These differences were significant in all the scales (Table 10); thus, both versions discriminated between these groups in all the scales.

**Table 8:** Comparison of the 7 scales of the Spanish and Catalan versions of the eHealth Literacy Scale across educational levels.

	Spanish version				Catalan version		
	Primary school education (n=97), mean (SD)	University education (n=151), mean (SD)	*P* value	Primary school education (n=90), mean (SD)		University education (n=140), mean (SD)	*P* value
Using technology to process health information	1.88 (0.56)	2.93 (0.47)	<.001	1.55 (0.57)		2.93 (0.57)	<.001
Understanding of health concepts and language	2.11 (0.38)	3.03 (0.44)	<.001	1.85 (0.49)		2.97 (0.47)	<.001
Ability to actively engage with digital services	2.00 (0.61)	3.12 (0.45)	<.001	1.83 (0.65)		3.15 (0.52)	<.001
Feel safe and in control	2.22 (0.42)	2.99 (0.52)	<.001	2.06 (0.57)		2.94 (0.61)	<.001
Motivated to engage with digital services	1.89 (0.47)	2.99 (0.47)	<.001	1.56 (1.55)		2.91 (0.57)	<.001
Access to digital services that work	2.04 (0.42)	2.83 (0.47)	<.001	1.73 (0.50)		2.89 (0.51)	<.001
Digital services that suit individual needs	1.91 (0.53)	3.00 (0.51)	<.001	1.62 (0.63)		2.92 (0.59)	<.001

**Table 9:** Comparison of the 7 scales of the Spanish and Catalan versions of the eHealth Literacy Questionnaire across socioeconomic levels.

	Spanish version				Catalan version		
	Low level (n=92), mean (SD)	High level (n=89), mean (SD)	*P* value	Low level (n=91), mean (SD)		High level (n=99), mean (SD)	*P* value
Using technology to process health information	2.20 (0.70)	3.00 (0.45)	<.001	2.19 (0.74)		2.87 (0.68)	<.001
Understanding of health concepts and language	2.37 (0.55)	3.02 (0.41)	<.001	2.35 (0.67)		2.93 (0.59)	<.001
Ability to actively engage with digital services	2.32 (0.72)	3.17 (0.44)	<.001	2.45 (0.83)		3.12 (0.61)	<.001
Feel safe and in control	2.42 (0.56)	2.99 (0.50)	<.001	2.37 (0.76)		2.90 (0.66)	<.001
Motivated to engage with digital services	2.21 (0.64)	3.07 (0.39)	<.001	2.15 (0.80)		2.89 (0.68)	<.001
Access to digital services that work	2.25 (0.54)	2.91 (0.42)	<.001	2.21 (0.68)		2.81 (0.64)	<.001
Digital services that suit individual needs	2.17 (0.66)	3.06 (0.42)	<.001	2.14 (0.84)		2.90 (0.67)	<.001

**Table 10:** Comparison of the 7 scales of the Spanish and Catalan versions of the eHealth Literacy Questionnaire across levels of use of technology.

	Spanish version				Catalan version		
	Never or occasionally (n=86), mean (SD)	Continuous (n=90), mean (SD)	*P* value	Never or occasionally (n=57), mean (SD)		Continuous (n=95), mean (SD)	*P* value
Using technology to process health information	1.94 (0.63)	3.06 (0.52)	<.001	1.93 (0.84)		3.07 (0.61)	<.001
Understanding of health concepts and language	2.23 (0.47)	3.04 (0.52)	<.001	2.13 (0.74)		3.01 (0.57)	<.001
Ability to actively engage with digital services	2.06 (0.65)	3.31 (0.54)	<.001	2.00 (0.89)		3.23 (0.63)	<.001
Feel safe and in control	2.35 (0.55)	3.03 (0.59)	<.001	2.29 (0.69)		3.00 (0.73)	<.001
Motivated to engage with digital services	1.99 (0.58)	3.08 (0.58)	<.001	1.89 (0.81)		3.00 (0.64)	<.001
Access to digital services that work	2.09 (0.48)	2.93 (0.55)	<.001	1.99 (0.72)		2.95 (0.62)	<.001
Digital services that suit individual needs	1.97 (0.61)	3.08 (0.56)	<.001	1.88 (0.84)		3.07 (0.67)	<.001

## Discussion

### Principal Findings

The main objective of this study was to undertake the translation and cultural adaptation of the eHLQ and gather evidence of the psychometric quality of the eHLQ in a large stratified sample of the general population in Spain, creating a parallel process for 2 of Spain’s official languages: Spanish and Catalan.

Overall, the new versions of the eHLQ in Spanish and Catalan were found to be highly coherent with the original validation of the English and Danish versions [22]. As shown by the simultaneous process carried out for the original versions to maximize comparability over linguistic differences, the Spanish and Catalan versions present similar patterns that were confirmed through measurement invariance in 2 different language versions within the same sociocultural context.

### Translation and Cultural Adaptation

The Translation Integrity Procedure [33] provided common ground and facilitated iterative discussions among the professional translators, the authors of the original questionnaire, and the authors of this study, leading to a final consensus that ensured that the translated versions maintained the same semantic and conceptual orientations as the original version [45]. Furthermore, this process generated some interesting insights and qualitative results regarding the conceptualization of the instrument and its equivalence in a context of different languages [34].

The cognitive interviews, which provided validity evidence based on the test content and the response process, showed that some of the items could be misunderstood in the new translated versions, and it was difficult to reach a consensus to overcome the cultural adaptation. This was particularly relevant for item eHLQ26 (*I use measurements about my body...*) as the term “measurements” in this setting is mainly associated with height and weight in both Spanish and Catalan. Thus, the wording of this item had to be changed slightly to “I use data on how my body works,” and some specific examples of this concept were included in the instructions page with the terms used in the questionnaire. Although the difficulties with the concept of measurements (including professional and fitness-tracking devices) can be related with the low indicator for Spain in a European survey [7], this item has also been problematic in previous studies [15,24,26]. For example, in the Australian and Taiwanese populations, this item had low factor loadings, and in the Dutch population, it had residual correlation issues. In this study, this item presented high factor loadings in both versions.

Along the same lines, other groups of examples of terms related to “communication and sharing of information” (both with professionals and other people) were also included to further clarify the intent of items eHLQ20 (*I use technology to share information...*) and eHLQ27 (*Technology improves my communication...*)*. *In fact, although recent reports and surveys show that Spain can be considered one of the leading countries in the use of digital technologies related to health [8,10], it seems that the concepts of “eHealth,” “digital health,” and “digital health services” are not well understood by the general population. Despite the fact that citizens are increasingly using them, there is little familiarity with these terms outside professional circles, highlighting the need for an instrument such as the eHLQ to assess the readiness of the population and help increase the adoption and use of digital health services.

Reinforcing the instructions page with more information forms part of the cultural adaptation process, and the eHLQ counts on this. The Spanish and Catalan versions of the eHLQ took advantage of this possibility to add more information. However, it did not provide further recommendations on their use, administration, or interpretation, as do other versions [26].

### Quantitative Validity Evidence

Results for both the Spanish and Catalan versions showed means around 2.5 points, with the 7 scales showing a similar deviation pattern. The values were in a similar range to that of the values obtained in the studies in Denmark (between 2.42 and 2.97) [22], Sweden (between 2.58 and 3.04) [25], and Taiwan (between 2.37 and 3.08). In all 3 cases, scale 2 (*Understanding of health concepts and language*) had the highest score, and scales 7 (*Digital services that suit individual needs*; for the Danish and Swedish versions) and 6 (*Access to digital services that work*; for the Mandarin version) had the lowest mean.

The 7-factor correlated model presented adequate GOFIs both in the Spanish and Catalan versions. Moreover, measurement invariance was found between both forms for the factor loadings. The CFA 1-factor model for each scale also fit well considering CFI and TLI, and all the items presented a strong factor loading within their respective scales. In this model, the RMSEA values had good results for some of the scales in the Spanish version, although this index can be problematic for assessing models with small df, as in this study [24].

According to the factor loadings, all the items were around the constraint value of each scale. In both versions, the scale with the highest loadings was scale 4 (*Feeling safe and in control*), as mentioned in recent surveys in Spain as the key element for the implementation of a digitalized and efficient health system [8,9]. For the Spanish version, almost all items had factor loadings of >0.80 except for 2 items of scale 6: eHLQ9 (*r*=0.77; *My health care providers deliver services that I can access through technology*) and eHLQ16 (*r*=0.78; *My health data are available to me wherever I am*). Item eHLQ9 was also the one with the lowest factor loading in the Catalan version. This cannot be considered a low factor loading as these results are in line with those of the original version, where these items showed the lowest factor loadings (0.44 and 0.43, respectively) [22]. Ensuring access and universal coverage for digital health systems, represented by scale 6, is still a major challenge for individuals to process and understand.

There was a high correlation between the dimensions, especially for factors 1 and 5 (*Using technology to process health informatio*n and *Being motivated to engage with digital services*), both in the Spanish and Catalan versions (*r*=0.90 in both cases). These values resemble the findings for the original version, which the authors attributed to the result of being located along the same causal path [22]. Both versions showed other high correlations between factors 5 and 7 (*r*=0.92 in Spanish and *r*=0.89 in Catalan), perhaps suggesting that motivation and engagement regarding using digital health services are related to the perception that those services suit the users’ specific needs and preferences.

To explore convergent and discriminant validity evidence, the eHEALS [12] was also administered, which is a good measure of some elements of digital health literacy. It has been adapted and has shown evidence of psychometric quality in Spanish in a sample of university students [36]. Mean scores were similar in the Spanish (27.35, SD 8.40) and Catalan (27.80, SD 7.08) versions and slightly lower than those in a study on the use of the internet and digital literacy among Spanish nurses, with a mean of 32.44 [46]. Moderate to low correlations of the eHEALS were found with the different eHLQ scales, as expected because of their different theoretical models [12,21]. This was an expected result given that, even though the eHEALS is still a widely used tool, it does not provide sufficient understanding of the individual’s interaction with digital services and technology or the user’s experience of engaging with the system, which are all covered by the eHLQ’s dimensions. For both translated versions of the eHLQ, the lowest correlation with the eHEALS was found for scale 4 (*Feel safe and in control*; *r*=0.56 in Spanish and *r*=0.43 in Catalan), probably because all the items within this scale relate to the ownership of personal data and their secure access, which are elements of a dimension not included at all in the eHEALS. Lower correlations were expected between the eHEALS and scales 3, 4, 5, 6, and 7, and discriminant evidence was searched for. The high correlation among the other subscales could be explained by the low discrimination among the subscales of the eHLQ, as was also the case in previous studies and as has been described previously.

As for internal consistency reliability, adequate values were found for all the scales even considering the minimal number of items (4-6) per scale, with Cronbach α and categorical ω values of >0.80 (and almost all >0.90) in all the scales in both the Spanish and Catalan versions. These values confirm that the selected items are consistent in each scale in both translated versions and are in line with the original results of the Danish validity testing study [22], with internal consistency values ranging from 0.77 to 0.86, and with the translation and adaptation to the Mandarin language [24], with values ranging from 0.75 to 0.95. In both studies, the lowest internal consistency values were also found for scale 2. Although the Swedish version [25] shows higher values (ranging from 0.82 to 0.92), scale 2 (together with scale 6) also shows the lowest internal consistency values.

Moreover, reliability was considered good as all the scales in both versions had α and ω values of >0.80, with values close to 0.90.

In both the Spanish and Catalan versions, all the scales showed the sensitivity to discriminate among known groups according to their educational and socioeconomic level and their self-categorization as technology users. However, results must be interpreted with caution as measurement invariance across the groups could not be studied due to sample size limitations. This is a relevant issue as a previous study in a Dutch sample could not establish full invariance among the different groups [26]. The differences were statistically significant for all the scales in these 3 known groups. Thus, people with a primary school education, lower socioeconomic status, or occasional use of technology scored lower than those with university studies, higher socioeconomic status, or continuous use of technology. This was apparent in all the scales and in both the Spanish and Catalan versions, with similar patterns of dispersion. Although the difference among the groups reveals the discrimination power of both versions of the questionnaire, this finding has to be taken with caution as it may also be an artifact due to the large samples and the dispersion shown by the scores.

Finally, a tendency to mark “disagree” rather than “strongly disagree,” especially for those groups with lower socioeconomic status, was revealed across the scales. This might reflect some respondents’ attempts to hide a lack of comprehension or the inappropriateness of the questions to their particular situation, prompting them to provide more socially desirable answers. The possibility was discussed of including some previous instructions to encourage people to provide an answer for all the items and answer “strongly disagree” in cases in which the question was not clearly understood or did not apply to their own situation. However, as answers should not be suggested to respondents under any circumstances, a general recommendation designed to encourage people to respond as well as they could was included, informing them that there were no correct or incorrect answers. In relation to that, the questionnaire raises aspects linked to digital health (eg, services and devices) that the Spanish and Catalan populations, even as educated and digital users, do not recognize in their current daily lives. Considering the rapid development of digital health in Spain, this will no longer be the case in the near future. For this reason, future testing studies of the eHLQ will probably not require such an extensive introduction to the questionnaire as the one that was included as a key element in the cultural adaptation of the instrument in this study.

### Strengths and Limitations

The translation and cultural adaptation of an instrument into 2 languages simultaneously is a strength of this study because they provide a tool with psychometric guarantees to assess health literacy. In addition, the stratified sample of the Spanish- and Catalan-speaking populations in terms of gender, age, and place of residence is another important strength that makes the eHLQ a good instrument to use in community and population surveys.

However, all the interviews were conducted using a digitally administered questionnaire, and therefore, all the respondents needed some basic level of digital skills, which may have excluded some people with no internet access or no basic skills from the sample. To overcome this limitation, the sample was balanced by conducting 200 (n=100, 50% for each version) face-to-face interviews in community settings targeted at people with primary school studies and of a low socioeconomic level, and 80% (60/200) of them were aged >60 years. In this sense, it is also important to note that, according to the self-categorization scale regarding technology use, the total sample included respondents who reported that they never or occasionally used technology (54/400, 13.5% in the Spanish version and 27/400, 6.7% in the Catalan version).

Regarding future studies, although the sample in this study was large and covered a wide range of sociodemographic, academic, and technology use profiles, it was not free of voluntary reporting and self-reporting biases. Further studies are suggested involving participants with different health conditions to provide evidence of the psychometric quality of the instrument in different contexts of use.

Future studies with larger sample sizes should consider testing for measurement invariance across groups to test whether group differences are due to measurement issues or real differences. Finally, it would be interesting to consider the development of further versions covering Basque and Galician, which are also spoken in Spain.

### Conclusions

This study demonstrates that the translated and adapted versions of the eHLQ in Spanish and Catalan are compatible with the original’s construct and item intent and maintain proper psychometric properties. The eHLQ provides a tool in Spain for researchers, policy makers, and health service managers to explore people’s needs, skills, and competencies and obtain interesting insights into their interactions and engagement regarding their own experiences with digital health services, especially in the context of digital health growth in Spain.

## Abbreviations

CFA: confirmatory factor analysis
CFI: comparative fit index
eHEALS: eHealth Literacy Scale
eHLQ: eHealth Literacy Questionnaire
GOFI: goodness-of-fit index
HLS-EU-Q: European Health Literacy Survey Questionnaire
RMSEA: root mean square error of approximation
TLI: Tucker-Lewis index

## Data Availability

The Catalan and Spanish versions of the eHealth Literacy Questionnaire are available upon request. Please contact Centre for Global Health and Equity at the Swinburne University of Technology [47] for further information. The data sets generated and analyzed during this study are available from the corresponding author on reasonable request.
